# Interferon-Alpha Mediates Restriction of Human Immunodeficiency Virus Type-1 Replication in Primary Human Macrophages at an Early Stage of Replication

**DOI:** 10.1371/journal.pone.0013521

**Published:** 2010-10-20

**Authors:** Kelly M. Cheney, Áine McKnight

**Affiliations:** Barts and The London School of Medicine and Dentistry, Queen Mary University of London, London, United Kingdom; University of Minnesota, United States of America

## Abstract

Type I interferons (IFNα and β) are induced directly in response to viral infection, resulting in an antiviral state for the cell. *In vitro* studies have shown that IFNα is a potent inhibitor of viral replication; however, its role in HIV-1 infection is incompletely understood. In this study we describe the ability of IFNα to restrict HIV-1 infection in primary human macrophages in contrast to peripheral blood mononuclear cells and monocyte-derived dendritic cells. Inhibition to HIV-1 replication in cells pretreated with IFNα occurred at an early stage in the virus life cycle. Late viral events such as budding and subsequent rounds of infection were not affected by IFNα treatment. Analysis of early and late HIV-1 reverse transcripts and integrated proviral DNA confirmed an early post entry role for IFNα. First strand cDNA synthesis was slightly reduced but late and integrated products were severely depleted, suggesting that initiation or the nucleic acid intermediates of reverse transcription are targeted. The depletion of integrated provirus is disproportionally greater than that of viral cDNA synthesis suggesting the possibility of a least an additional later target. A role for either cellular protein APOBEC3G or tetherin in this IFNα mediated restriction has been excluded. Vpu, previously shown by others to rescue a viral budding restriction by tetherin, could not overcome this IFNα induced effect. Determining both the viral determinants and cellular proteins involved may lead to novel therapeutic approaches. Our results add to the understanding of HIV-1 restriction by IFNα.

## Introduction

Type I interferon (IFN) α and β can be induced directly in response to viral infection and trigger the transcription of a diverse range of IFN-stimulated genes (ISGs) through activation of the Jak-STAT (signal transducer and activator transcription) pathway [1]. This establishes an antiviral state in target cells. However, most viruses can still replicate and cause disease *in vivo*, having evolved strategies to at least partially avoid or inhibit the IFN response. The efficiency by which the virus manages to circumvent the antiviral actions of IFN is important in the establishment of a productive infection.

Multiple clinical trials have tested the ability of IFNα to restrict HIV-1 replication or slow disease progression [2], [3], [4], [5], [6]. Overall, compared with the efficacy seen in treatment of viral infections such as Hepatitis C, the *in vivo* effects of IFNα in HIV-1 infected patients were modest at best, with conflicting results for negative outcomes such as toxicity, antiretroviral treatment failure and progression of HIV-1 disease. This was somewhat surprising as *in vitro* studies in both cell lines and primary human cells showed that IFNα is a potent inhibitor of HIV-1 infection, particularly in the early stages [7], [8], [9], [10], [11], [12], [13], [14]. The role of IFNα in HIV-1 infection is incompletely understood, however results from this and other studies suggest that IFNα may regulate the expression of a restriction factor/s able to specifically inhibit the replication of HIV-1.

More recent work has linked the ability of IFNα to inhibit HIV-1 infection in cell lines with a cellular membrane protein CD317 (tetherin) and known HIV-1 restriction factor APOBEC3G [15], [16], [17]. Tetherin is upregulated by IFNα and inhibits the release of newly assembled virions in cell lines. IFNα also upregulates the level of APOBEC3G in primary human macrophages. The enzymatic activity of APOBEC3G leads to the degradation of HIV-1 DNA [18], [19]. To mediate this effect however, APOBEC3G must be incorporated into the virion and thus is only able to restrict the establishment of new rounds of infection. Incorporation of APOBEC into virions is prevented by the viral accessory protein, Vif.

Here, we confirm previous studies in MDM and extend them to describe a dramatic reduction in HIV-1 infection by IFNα in primary human macrophages but interestingly not dendritic or T cells derived from the same donors. Our experiments support data indicating that IFNα acts at an early stage in the virus life cycle, upon establishment of infection. Our analysis of integrated proviral levels suggests the possibility of an additional target between the completion of reverse transcription (RT) and integration. The inhibition of replication is not due to IFNα-induced action of tetherin or APOBEC3G. Rather we suggest IFNα inhibits HIV-1 through an as yet unknown cellular pathway, possibly targeting the viral *gag/pol*, the reverse transcription process itself or RT intermediates. Identification of the viral and cellular factors involved in this response may provide novel therapeutic approaches without the historical negative outcomes of IFNα.

## Results

### IFNα dramatically inhibits HIV-1 replication in MDM but not PBMC/MDDC

**Figure 1:**
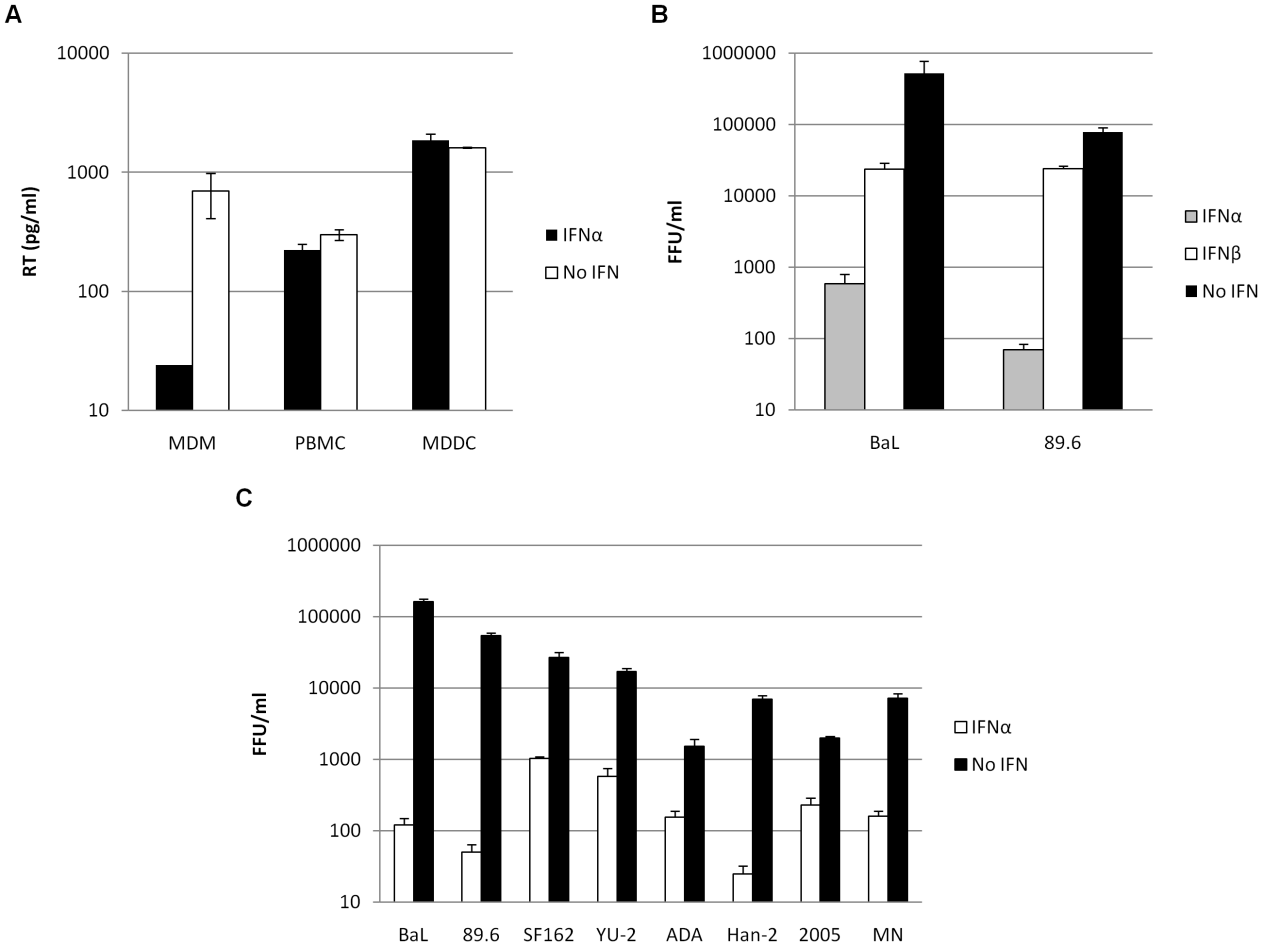
IFNα potently inhibits HIV-1 replication in MDM but not PBMC/MDDC. (a) MDM, PBMC and MDDC were treated with 500 IU/ml IFNα 24 hr prior to challenge with HIV-1 89.6. Culture supernatants were assayed after 4 days for levels of RT by ELISA (pg/ml). (b & c) MDM were treated with 500 IU/ml IFNα or β 24 hr prior to infection with a panel of replication competent HIV-1. Infected foci were counted after 4 days. Fold reduction is the ratio of FFU/ml of no IFN compared to IFNα treated cells. Error bars represent SD of one representative experiment.

MDM, PBMC and MDDC were infected with HIV-1 89.6 in the presence or absence of IFNα. Viral replication was determined after 4–7 days following analysis of reverse transcriptase levels in the culture supernatants. IFNα was shown to inhibit HIV-1 replication in MDM. In contrast pretreatment of PBMC and MDDC with IFNα did not show any effect on the production of new virions (Fig. 1a). This suggests that IFNα can mediate a restriction to HIV-1 replication in a cell specific manner.

The general antiviral roles of the type I IFNs are well described, and to determine the relative ability of members of this IFN family to inhibit HIV-1, MDM were challenged with replication competent HIV-1 strains BaL and 89.6 in the presence or absence of IFNα or IFNβ. The level of HIV-1 infection was determined after 4 days by *in situ* intracellular p24 staining and enumeration of FFU/ml. Fold reduction is the ratio of FFU/ml of no IFN compared to IFNα treated cells. Replication in MDM by these HIV-1 isolates is dramatically inhibited (up to 1000-fold) when these cells are pretreated with IFNα (Fig. 1b). While it is known that IFNβ performs a unique role in viral infection and is essential for a fully effective general antiviral response, the potency of HIV-1 restriction was much less when MDM were pretreated with IFNβ. These data are consistent with previous reports that HIV-1 is susceptible to IFNα mediated inhibition [9], [10]. Results for IFNβ have not been previously reported. In this study we specifically investigate IFNα, and its ability to mediate restriction of HIV-1 infection.

Further analysis was then performed on an extended panel of full length HIV-1 isolates. The well characterised macrophage- (BaL, YU-2) and dual-tropic (89.6) HIV-1 strains along with several primary isolates were used. HIV-1 isolates were dramatically inhibited (up to 1000-fold) when these cells were pretreated with IFNα (Fig. 1c). The extent of inhibition across the panel of viruses tested was variable (10-1000-fold), indicating that some isolates are less sensitive to the antiviral action of IFNα and suggesting the evolution of viral escape mechanisms.

### IFNα inhibits the establishment of infection and acts via unknown cellular factor/s

**Figure 2:**
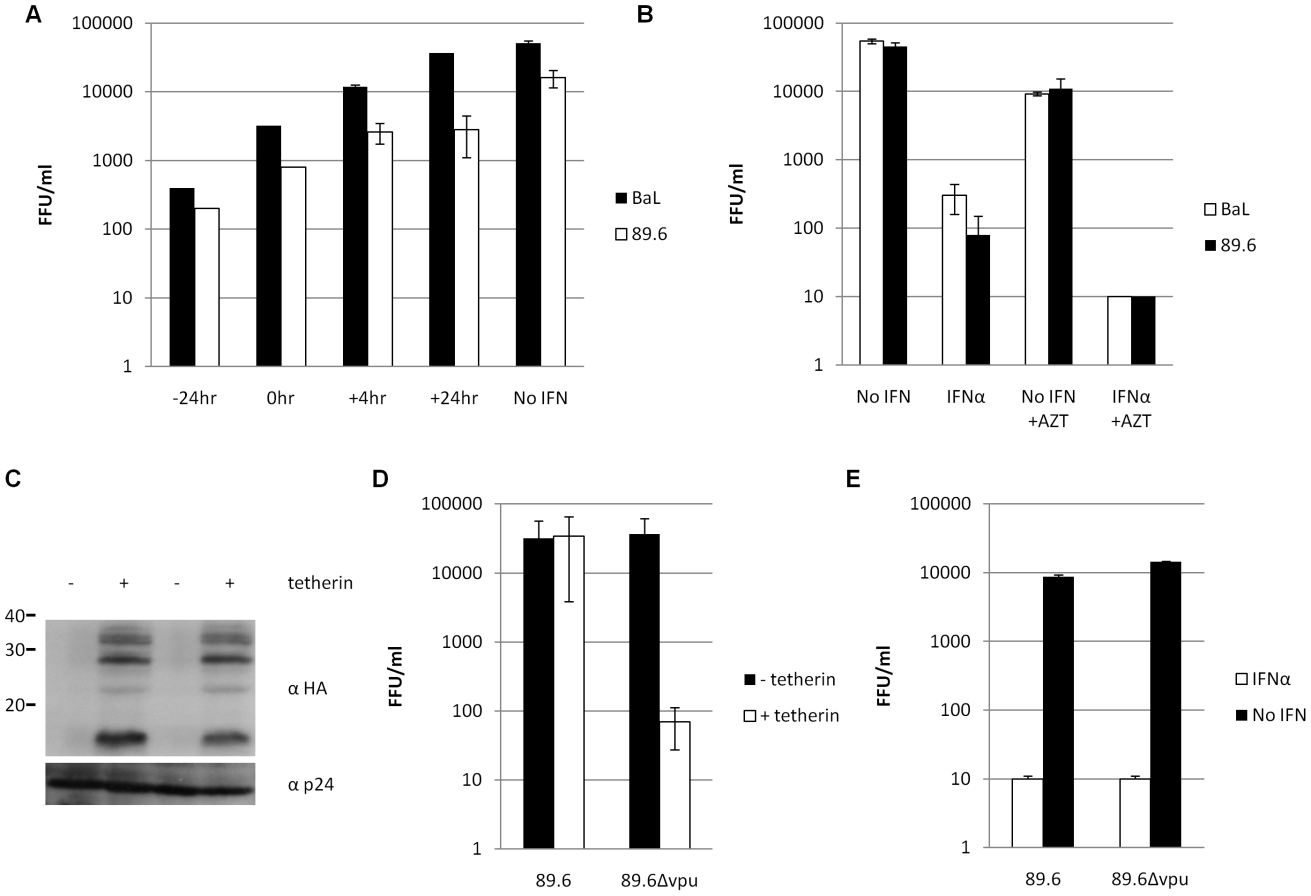
IFNα inhibits the establishment of infection and acts via unknown cellular protein/s. MDM were treated with 500 IU/ml IFNα 24 hr prior to infection with replication competent HIV-1 89.6 and BaL. Infected foci were counted after 4 days. (a) IFNα was added at various time points prior to, at or after infection. (b) AZT (final concentration 100 nM) was added to cultures 24 hr post infection to block second round infection. (c) HEK 293T cells were co-transfected with pcDNA3.1-HA-tetherin and either WT or Δ*vpu* 89.6 molecular clones. Cells were lysed and the presence or absence of tetherin was confirmed by Western blot using an anti-HA Ab. Tetherin is a 30–36 kDa protein that migrates as several species by SDS-PAGE, as a result of post-translational modifications [15]. The levels of p24 protein were monitored as a loading control. (d) Supernatants from transfected HEK 293T cells were serially diluted and used to infect NP2-CD4-CXCR4 cells. Virus titres were determined after 48 hr. (e) MDM were challenged with tetherin resistant (WT) and tetherin sensitive (Δ*vpu*) HIV-1 molecular clones ± IFNα. Data is presented as mean ± SD.

MDM cultures were treated with IFNα at various times prior to, at and post infection with HIV-1 strains 89.6 and BaL, previously shown to be highly sensitive to its inhibitory effects. The most potent restriction was observed when IFNα was present before the cells were infected (Fig. 2a). IFNα was still seen to have a modest effect on replication when added at the same time as the virus, but this soon decreased to low/negligible levels if IFNα was introduced after first round infection was complete (+24 hr). These results show that suitable expression levels of antiviral proteins or non-translated RNA can take at least 4 hr following induction by IFNα (up to 24 hr for maximum effect) and suggest that the resultant antiviral state is most effective at inhibiting the initial establishment of infection.

APOBEC3G is a well known IFNα responsive gene that inhibits HIV-1 replication at an early post entry stage (reviewed in [20]). The action of APOBEC3G on nascent DNA requires its presence in virions made by the producer cell. HIV-1 Vif counteracts the action of APOBEC3G by preventing its incorporation into virions. Here it is unlikely that APOBEC3G restriction is responsible for the IFNα mediated inhibition because the HEK 293T producer cells are APOBEC3G^-^ [21]. Furthermore virion incorporation of APOBEC3G is unlikely because all viruses are Vif^+^. It could be argued however that the higher cellular levels of APOBEC3G following IFNα induction overcome Vif mediated exclusion. This could result in APOBEC3G being packaged into virions after first round infection and contributing to the total inhibition. To exclude this possibility, experiments were performed in which the RT inhibitor AZT was introduced 24 hr post infection, to block late stages and second round infection (Fig. 2b).

If late events or APOBEC3G contributed to the inhibition induced by IFNα, we would expect a relative reduction of IFNα mediated inhibition in cells treated with AZT. In MDM infected with HIV-1 BaL and 89.6, no difference in the ability of IFNα to restrict infection was observed in the presence or absence of AZT (Fig. 2b), confirming the notion that IFNα-induced restrictions act at an early stage of viral replication.

A role has recently been attributed to IFNα in the regulation of tetherin (CD317, BST-2), a cell surface molecule capable of inhibiting viral production. The inhibition is overcome by HIV-1 Vpu [15], [16].

We determined whether the IFNα mediated restriction described here could be due to the action of tetherin. We engineered an HIV-1 molecular clone 89.6 with a premature stop codon in *vpu* (89.6Δ*vpu*). We first confirmed that the 89.6Δ*vpu* construct was susceptible to tetherin: HEK 293T cells were cotransfected with HA-tagged tetherin and either wild type (WT) or Δ*vpu* HIV-1 89.6 molecular clones. Tetherin and viral protein expression was confirmed by Western blot (Fig. 2c). The titre of supernatants collected from these cultures was determined confirming that tetherin was capable of inhibiting virus release in these assays (Fig. 2d). The presence of tetherin had no effect on WT 89.6 virus titre however, as expected, it was able to reduce the titre of 89.6Δ*vpu* at least 100-fold.

WT and Δ*vpu* HIV-1 strains were then used to challenge MDM in the presence or absence of IFNα. The results shown in Fig. 2e demonstrate that Vpu is not required for the IFNα restriction, and thus neither tetherin nor Vpu are involved in the IFNα induced restriction in MDM. Supporting this, a reduction was also observed in the levels of RT detected in the supernatants of MDM, PBMC and MDDC cultures when challenged with 89.6Δ*vpu* compared with WT 89.6 but no difference in the ability of IFNα to restrict HIV-1 replication was observed (data not shown). The IFNα mediated restriction described in this study therefore, is distinct from the effects of tetherin and Vpu.

### IFNα inhibits HIV-1 at an early post entry stage in the virus life cycle

To probe the mechanism behind the inhibition of HIV in MDM, we sought to determine the stage of the virus life cycle at which IFNα was effective.

**Figure 3:**
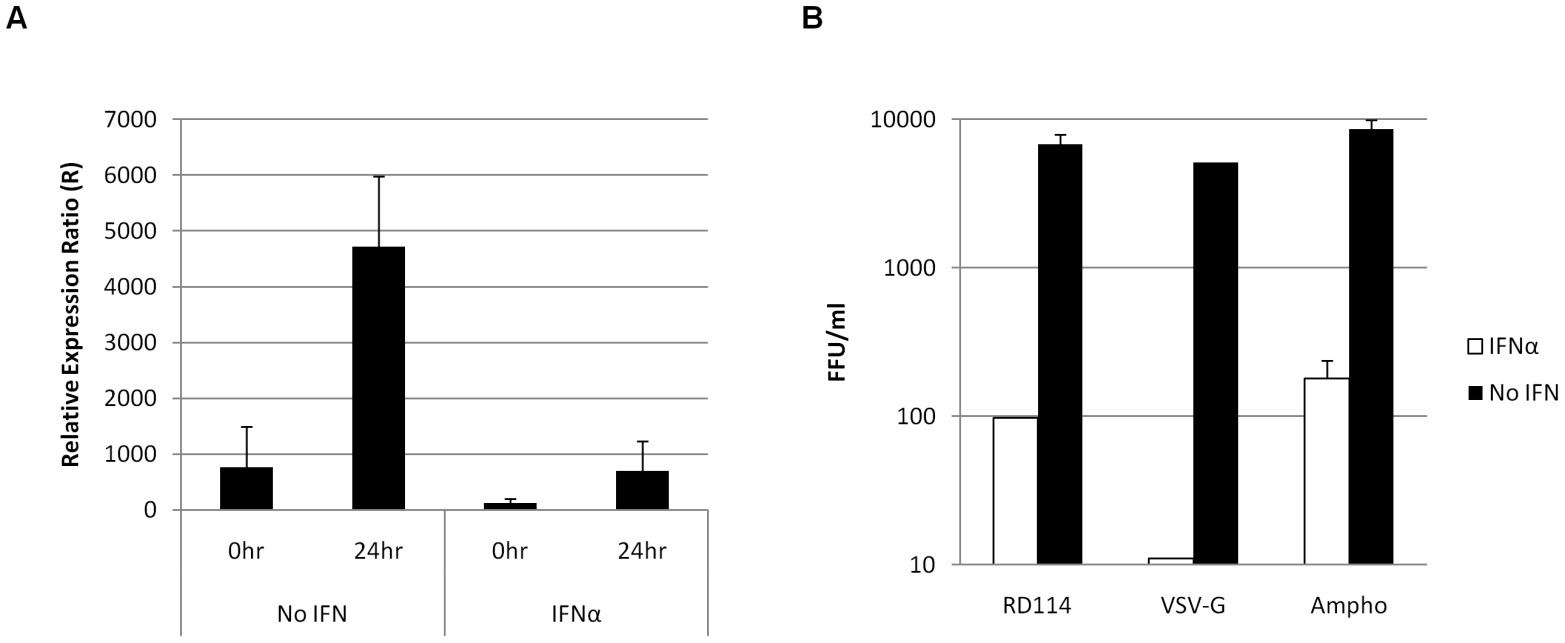
IFNα inhibits HIV-1 at an early post entry stage in the virus life cycle. (a) MDM were treated with 500 IU/ml IFNα 24 hr prior to challenge with HIV-1 BaL. Cells were collected 0 and 24 hr post infection and total RNA was extracted. RT-qPCR was used to detect late HIV-1 products and results were normalised to GAPDH cDNA and compared with uninfected controls. Error bars represent SD of two independent experiments. (b) HIV-1 89.6Δ*env* was pseudotyped with VSV-G, RD114 and Ampho MLV envs and used to challenge MDM. Infected foci were counted after 4 days and are presented as mean ± SD.

The lack of p24 protein in the MDM detected by staining for FFU indicated that the IFNα mediated block of viral replication was in the early part of the life cycle and at least prior to protein translation. Furthermore to support this we isolated mRNA from MDM infected with HIV-1 BaL and performed qRT-PCR to determine whether the quantity of HIV-1 transcripts produced in the presence of IFNα was affected. HIV-1 mRNA levels were negligible in the presence of IFNα (Fig. 3a). These results support the observations in Fig. 1c that IFNα acts at an earlier stage in the viral life cycle.

To expand on these results and characterise the viral determinants of IFNα restriction, MDM were infected with HIV-1 pseudotypes carrying various retroviral and other envelopes. Significant restriction was seen in all cases, most notably for HIV-1 cores pseudotyped with VSV-G, for which 4-5-log reductions in replication were observed (Fig. 3b).

Viruses pseudotyped with retroviral envelopes from feline endogenous virus (RD114) and amphotrophic murine leukaemia virus (Ampho) enter the cell following binding to the receptors RDR and PiT2 respectively, while VSV-G triggers entry by clathrin-mediated endocytosis through a ubiquitously expressed glycolipid. Therefore these results show that IFNα mediated restriction of HIV-1 is not dependent on the Env-host cell interaction. IFNα may mediate a restriction that targets the viral *gag-pol*.

### Post entry restriction affects the kinetics of late HIV-1 RT products in MDM but not PBMC/MDDC

**Figure 4:**
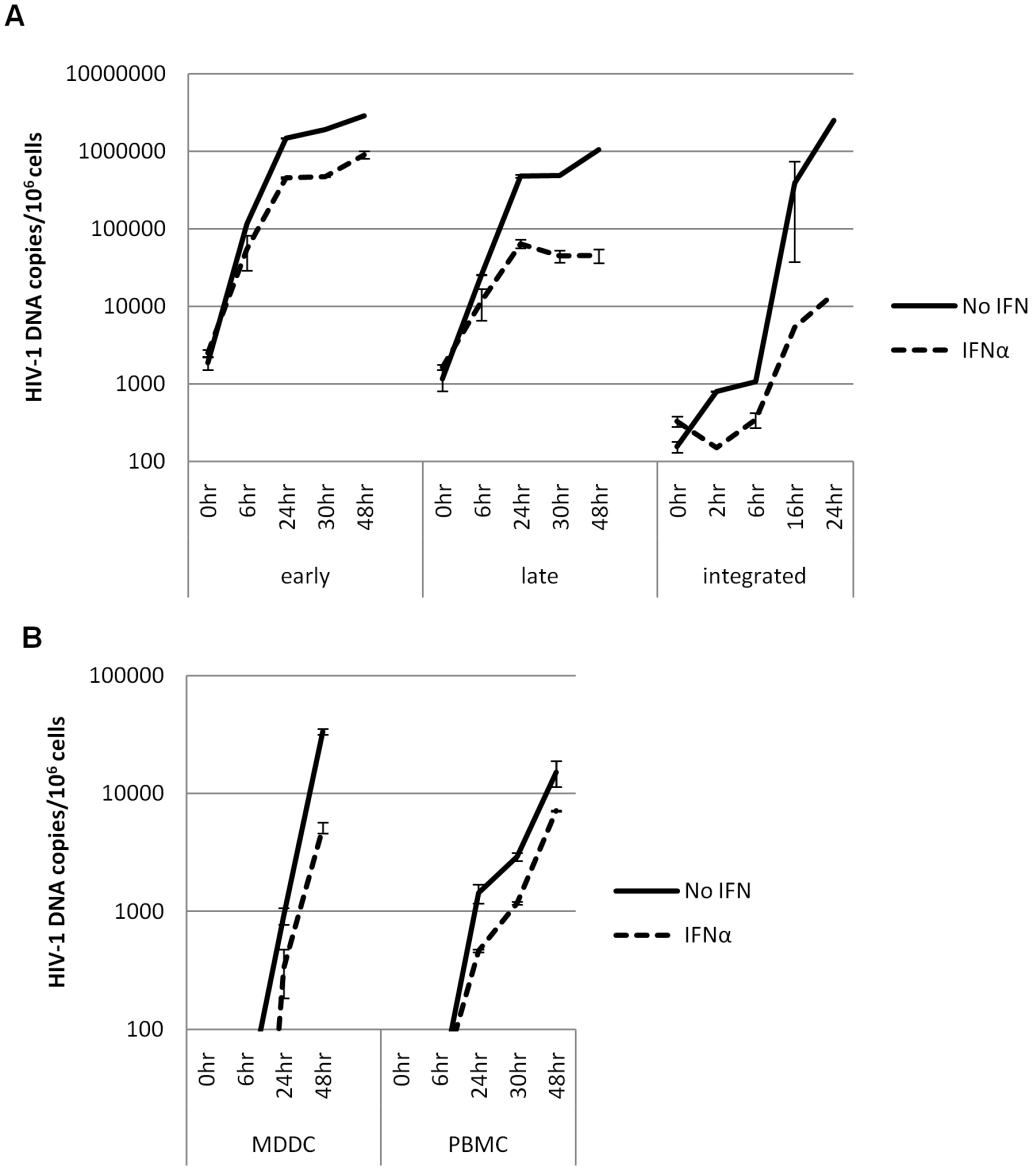
Post entry restriction affects the kinetics of late HIV-1 RT products and integration in MDM but not PBMC/MDDC. (a) MDM and (b) PBMC and MDDC were treated with 500 IU/ml IFNα 24 hr prior to challenge with HIV-1 BaL. Cells were collected at various time points after infection and DNA was extracted. Early (MDM), late (MDM, PBMC & MDDC) and integrated (MDM) HIV-1 DNA was normalised to genomic GAPDH and copy number is presented per 10^6^ cells. Data is presented as mean ± SD and is from one representative experiment.

To identify more precisely the post entry point of the restriction induced by IFNα, qPCR analysis was performed on DNA extracted from HIV-1 89.6 infected MDM at various time points post infection. Early (negative strand strong stop, -sss), late (*gag*-LTR) and integrated (*Alu*-*gag*) HIV-1 products were quantified. The results in Fig. 4a confirm that inhibition occurs post entry, as HIV-1 DNA products are detected in the presence and absence of IFNα. No HIV-1 DNA products are detected in the presence of a CCR5 inhibitor (data not shown). Up to 6 hr after virus challenge the levels of both early and late HIV-1 DNA RT products are the same in the presence or absence of IFNα, indicating the same efficiency of viral entry. After 6 hr IFNα treated samples showed a delay in both RT products. This was more pronounced for late RT transcripts, and more so for integrated proviruses, of which a 100-fold decrease was observed following treatment with IFNα. Thus this IFNα-induced restriction acts after the initiation of RT, preventing the completion and/or integration of full length products.

We next compared the effect of IFNα on RT in PBMC and MDDC. As expected from the infectivity data in Fig. 1a the potency of any IFNα induced restriction(s) was much weaker in PBMC and MDDC compared to MDM following analysis of the kinetics of HIV-1 DNA production (Fig. 4b).

## Discussion

Here we show a potent (3-log) IFNα induced antiviral response against HIV-1 infection which is specific to MDM. The inhibition of replication occurs after the virus delivers its capsid into the cytoplasm, acting after initiation, but before completion of RT. We have excluded the involvement of previously described IFNα responsive genes tetherin or APOBEC3G.

The overall modest responses of HIV-1 infected patients to IFN therapy in past years [2], [3], [4], [5], [6] have forced researchers to investigate alternative strategies for the development of pharmaceuticals to control disease progression. Subsequently, while long observed, the inhibitory effects of IFNα on HIV-1 replication in primary cells are relatively poorly understood. Here we have taken further steps in identifying and characterising its specific action on HIV-1 infection of primary human macrophages.

It has previously been reported that IFNα preferentially inhibits HIV-1 replication in MDM compared with CD4^+^ T cells [10]. Here we confirm this observation using RT ELISA and real-time qPCR analysis (Fig. 1a and 4b). We build upon previous studies of this nature with analysis of MDDC, in which we show a profile of HIV-1 replication more similar to PBMC than MDM. Thus we confirm the strong antiviral response to IFNα to be cell type specific, which may contribute to the more refractory nature of macrophages to HIV-1 infection than T-cells *in vivo*.

qPCR analysis of DNA from various time points following HIV-1 infection of MDMs shows that, on top of an overall decrease in the total amount of detectable reverse transcribed HIV-1 DNA, IFNα acts at a point after the initiation of reverse transcription which specifically delays the completion of full-length preintegration genomes and significantly reduces the levels of integrated provirus. This is supported by our observation of the redundancy of HIV-1 Env in determining susceptibility to IFNα, which indicates that this effect is not dependent on the Env-host cell interaction. The restriction acts after the viral core is uncoated and released into the cytoplasm, suggesting that the HIV-1 *gag/pol*, the reverse transcription process and events leading to integration may be targeted.

Analysis of the levels of integrated viral DNA following treatment with IFNα shows a 100-fold decrease compared with untreated samples 24 hr post infection. Yet there is only a 10-fold decrease in late RT products which could point to the existence of additional blocks after reverse transcription but before completion of integration due to the upregulation and action of multiple ISGs.

As the experiments in this study were performed using replication competent viruses, it was important to determine the extent to which the overall inhibition described could be accounted for by the action of known HIV-1 specific IFNα-inducible proteins tetherin and APOBEC3G. We have excluded a role for either protein in this IFNα mediated antiviral effect. Tetherin was excluded following the comparison of WT (tetherin resistant) and Δ*vpu* (tetherin sensitive) HIV-1 viruses wherein no difference in the ability of IFNα to restrict was observed. Interestingly, in a recent report of potential IFNα-induced antiviral genes, tetherin was upregulated less than 2-fold in MDM [22].

Evidence for IFNα regulation of APOBEC3G as an important intracellular defence against HIV-1 infection is compelling. It is unlikely that the IFNα mediated restriction described in this study is due to APOBEC3G. All viruses were Vif^+^ and produced in the absence of APOBEC3G. However it could be argued that IFNα overcomes APOBEC3G sequestration by HIV-1 Vif, allowing enhanced APOBEC3G packaging into budding virions. Our data using AZT to inhibit subsequent rounds of infection demonstrates that this scenario is unlikely as there was no relative difference in the potency of IFNα mediated restriction in the presence of AZT.

Generally, the IFNs are known to have wide ranging protective immunomodulatory, antiproliferative and antiviral effects, and exert their activity through multiple pathways. Effector proteins and pathways induced by IFN include protein kinase regulated by RNA (PKR)/eukaryotic initiation factor 2α (eIF-2α) for mRNA translation inhibition, RNase L for RNA degradation, adenosine deaminase acting on RNA (ADAR1) for RNA editing and others such as protein GTPase Mx and nitric oxide synthetase (NOS) (reviewed in [23]). In the case of HIV-1 infection, IFNα can itself be regulated by cytokines such as IL-27 [24], and has been implicated in the antiviral actions of activator proteins such as tetherin [15] and APOBEC3G [17], [25]. In addition to these mechanisms, we describe a role for IFNα in the early stages of establishment of initial infection that is peculiar to primary MDM.

In the last few years, several groups have characterised the ability of the retroviral protein Vpx to rescue HIV-1 infection in MDM by overcoming a restriction imposed by an unknown antiviral factor in these cells [26], [27], [28]. More recently, one study has suggested that the cellular factor overcome by Vpx is IFNα-inducible, as introduction of Vpx into IFNα treated MDM prior to HIV-1 infection rescues the level of inhibition approximately 100-fold [29]. The phenotype of the Vpx-counteractable restriction bears many similarities to the IFNα mediated restriction described here, as the antiviral action is at an early stage in the viral life cycle and the accumulation of full length reverse transcripts is prevented.

It seems unlikely that the cellular restriction factors responsible for the Vpx-counteractable phenomenon and the inhibition described here are one and the same. A recent study demonstrates that primate and nonprimate lentiviruses containing Vpx are also susceptible to restriction by IFNα [14]. Together with the observation that Vpx is not able to completely rescue HIV-1 from IFNα mediated inhibition, and that it has been shown to have the same enhancement of viral infection in MDDC [30] does not completely explain the total impact of IFNα on HIV-1 replication and thus it is likely that other unidentified IFNα-inducible antiviral factors exist.

Macrophages have a key role in the success of the innate immune response. A recent paper shows that HIV-1 infected MDM fail to produce type I IFNα suggesting that HIV-1 can specifically interfere with the IFNα mediated response to establish infection *in vivo* [31]. If the cellular proteins involved in this interference can be determined and targeted, without the toxicities and negative disease outcomes of previous IFNα therapies, further rounds of macrophage infection may be eliminated. Additionally, cell-cell transmission and/or establishment of cellular reservoirs with the potential to generate drug resistant strains may be reduced, enhancing the effectiveness of current long-term ART regimens [32].

## Materials and Methods

### Cells

Buffy coats from seronegative donors were obtained from the National Blood Service (Brentwood, UK). Donors were anonymous and thus patient consent was not required. Peripheral blood mononuclear cells (PBMC) were prepared by density-gradient centrifugation (Lymphoprep, Axis-Shield). Monocyte-derived macrophages (MDM) were prepared by adherence as described previously [33]. Cells were plated at 2×10^6^ cells/ml and left to differentiate for 7–14 days in RPMI 1640/20% autologous human serum and 20 ng/ml macrophage colony stimulating factor (M-CSF; R&D Systems).

PBMC were cultured from the same donor at 2×10^6^ cells/ml in RPMI 1640, supplemented with 10% foetal calf serum (FCS; Biosera), 1% penicillin/streptomycin (PAA) and phytohemagglutinin (PHA-P, 0.5 µg/ml; Sigma). After 3–5 days, PHA-P was replaced by recombinant human interleukin-2 (rhIL-2, 20 U/ml; Invitrogen).

Monocyte-derived dendritic cells (MDDC) were isolated from PBMC using CD14^+^ MACS Microbeads (Miltenyi Biotech) and were cultured for 7 days in RPMI 1640/10% FCS, 1% penicillin/streptomycin, recombinant human interleukin-4 (rhIL-4, 50 U/ml; R&D Systems) and recombinant human granulocyte-macrophage colony stimulating factor (GM-CSF, 0.1 µg/ml; Sigma).

HEK 293T [34] cells were cultured in Dulbecco's modified Eagle's medium (DMEM; Invitrogen) with 10% FCS and 1% penicillin/streptomycin. DMEM for NP2-CD4-CXCR4 [35] cells additionally contained geneticin (G418, 1 mg/ml; Melford Laboratories) and puromycin (1 µg/ml; PAA).

### Plasmid constructs

HIV-1 89.6Δ*vpu* and 89.6Δ*env* were generated from the 89.6 molecular clone using overlap extension PCR. For 89.6Δ*vpu*, the fragments obtained with the primers 89.6-ERI-F/Vpu stop-XbaI-R (89.6-ERI-F 5′-GGAGTGGAAGCCTTAATAAGAATTCTGCA-3′, Vpu stop-XbaI-R 5′-GCTAATATTTGTCTAGAAAGTTATACATGTAC-3′) and Vpu stop-XbaI-F/89.6-StuI-R (Vpu stop XbaI-F 5′-GTACATGTATAACTTTCTAGACAAATATTAGC-3′, 89.6-StuI-R 5′- GATACCTTTGGACAGGCCTGTGTA-3′) were used as a template for another PCR using the primers 89.6-ERI-F/89.6-StuI-R. The resulting fragment was cloned at *Eco*RI and *Stu*I sites in the 89.6 molecular clone. For 89.6Δ*env*, the fragments obtained with the primers 89.6-ERI-F/Env stop-HIII-R (Env stop-HIII-R 5′-TCCTGATCTCCTTCAAGCTTTATGCCACTGTC-3′) and Env stop-HIII-F/89.6-StuI-R (Env stop HIII-F 5′-GACAGTGGCATAAAGCTTGAAGGAGATCAGGA-3′) were used as a template for another PCR using the primers 89.6-ERI-F/89.6-StuI-R. The resulting fragment was cloned at *Eco*RI and *Stu*I sites in the 89.6 molecular clone.

To generate the HA-tagged pcDNA3.1-tetherin construct, tetherin coding sequence was amplified using forward (5′-CACACACACACAGCGGCCGCATGTACCCATACGATGTTCCAGATTACGCTATGGCATCTACTTCGTATGACTA-3′) and reverse (5′-CACACACACACAGGATCCTCACTGCAGCAGAGCGCTGA-3′) primers from 293T cDNA and cloned in-frame at *Not*I and *Bam*HI sites in the pcDNA3.1(-) expression vector (Invitrogen).

### Primary HIV-1 stocks

2005 and HAN-2 were isolated as previously described [36]. All primary strains were minimally passaged in PBMC to expand virus stocks.

### Virus stocks

Virus stocks were prepared from infectious full-length and chimeric HIV-1 clones by polyethylenimine (PEI; Polysciences) transfection of HEK 293T cells. **Infectivity assays.** NP2-CD4-CXCR4 cells were seeded in 48-well trays at 1.5×10^4^ cells/well in 200 µl antibiotic-free DMEM. The following day viruses were added to the cells and incubated for 2 hr before the media was changed. Cells were cultured for 48 hr before infection was assessed following intracellular p24 staining.

MDM in 48-well trays (4×10^5^ cells/well) were infected with 500–5000 FFU viral stock (titred on NP2 cells) in 200 µl RPMI 1640/20% autologous serum. Media was replaced after 24 hr and virus production was detected after 4 days following intracellular p24 staining.

Time courses of virus infectivity of MDM, PBMC and MDDC were performed in 12- and 24-well trays. Cells were infected 3–5 days (PBMC) or 7 days (MDM and MDDC) post isolation. 500 IU/ml IFNα was added 24 hr prior to infection where appropriate. CCR5 coreceptor inhibitor SCH-D (10 nM) was added 1 hr before infection. MDM were challenged with 6000 focus forming units (FFU) virus/well in a total of 1 ml RPMI 1640/20% autologous serum while all PBMC or MDDC were initially pooled, pelleted and challenged with 250000 FFU virus. 2 hrs after infection these cells were divided into 24-well plates and made up to 1 ml in RPMI 1640/10% FCS supplemented with appropriate cytokines. At the collection of each time point, cells were washed twice with PBS.

### Determination of RT activity

Virus supernatants from infections were assessed for RT activity by RT-ELISA (Cavidi Tech Inc.).

### *In situ* immunostaining for p24 antigen

Infected cells were fixed with cold (−20°C) methanol:acetone (1∶1), washed with PBS then immunostained for p24 using mouse anti-HIV-1 p24 monoclonal antibodies EVA365 and 366 (NIBSC, UK) (diluted 1∶50), as previously described [37]. Infected cells were blue (regarded as foci of infection (FFU/ml)) and counted by light microscopy.

### Western blot

Western blots carrying lysed HEK 293T extracts were first incubated with primary antibody (rat anti-HA or human anti-p24) followed by the appropriate HRP-conjugated antibodies. Proteins were visualised using a chemiluminescence kit (ECL, Amersham Biosciences).

### First round *Alu-gag* PCR

DNA was extracted at various time points after infection with a QIAamp DNA Blood Mini Kit (QIAGEN). Integrated HIV-1 DNA was measured using a nested PCR protocol, as previously described [38]. Briefly, a standard curve was prepared using a 1∶1∶1 ratio of genomic DNA from chronically HIV-1 infected cell lines ACH-2 [39], 8E5/LAV [40] and OM-10.1 [41] in a background of HeLa genomic DNA (data not shown). First round products were amplified using 300 nM each primer (*Alu-*F 5′-GCCTCCCAAAGTGCTGGGATTACAG-3′; gag-R 5′-GTTCCTGCTATGTCACTTCC-3′) and approximately 50 ng total DNA in a 25 µl reaction. PCR conditions consisted of one cycle of denaturation (94° for 2 min), 39 cycles of amplification (94° for 15 sec, 50° for 30 sec, 68° for 8 min), and one cycle of 68° for 7 min. 5 µl of resulting PCR product was used as the template for measurement of integrated HIV-1 DNA in a nested real-time quantitative PCR.

### Real-time quantitative PCR for HIV-1 DNA

The isolated DNA was subjected to real-time quantitative PCR (qPCR) to determine the number of early (negative strand strong stop, -sss) and late (*gag*) transcripts present, normalised for cell number by genomic GAPDH. Integrated HIV-1 DNA copies were determined in a nested qPCR using 5 µl of first round product as template. Each 25 µl reaction contained the following components: 1× MegaMix-Gold PCR buffer (Microzone), 300 nM each forward primer (early 5′-GCTAACTAGGGAACCCACTGCTT-3′; late 5′-tgggcaagcagggagcta-3′; genomic GAPDH 5′-GAAGGTGAAGGTCGGAGT-3′; integrated 5′- TTAAGCCTCAATAAAGCTTGCC-3′), 300 nM each reverse primer (early 5′-CAACAGACGGGCACACACTACT-3′; late 5′-tcctgtctgaagggatggttgt-3′; genomic GAPDH 5′-CATGGGTGGAATCATATTGGAA-3′; integrated 5′- GTTCGGGCGCCACTGCTAGA-3′), 150 nM each probe (early 5′-CY3-AGCCTCAATAAAGCTTGCCTTGAGTGCTTC-BHQ2-3′; late 5′-6-FAM-aacgattcgcagttaatcctggcctgtt-BHQ1-3′; genomic GAPDH 5′-CY5-CAACGGATTTGGTCGTATTGGGCGC-BHQ2-3′; integrated 5′- CY3-CCAGAGTCACACAACAGACGGGCACA-BHQ2-3′) and approximately 500 ng total DNA (early, late, GAPDH) or 5 µl of first round *Alu-gag* PCR product (integrated). A standard curve was prepared with the NL4.3 molecular clone (early and late HIV-1 DNA) and HEK 293T genomic DNA (GAPDH) in a background of 200 ng salmon sperm carrier DNA (data not shown). PCR conditions consisted of one cycle of denaturation (95°C for 5 min) followed by 40 cycles of amplification (95°C for 15 sec, 60°C for 1 min). Data acquisition and analysis was performed using the ABI PRISM 7500 SDS software.

### cDNA synthesis

Total RNA was extracted from MDM using an RNeasy Plant Mini Kit (QIAGEN) and cDNA was synthesised with Superscript III First Strand Synthesis System (Invitrogen), according to manufacturer's instructions. The cDNA produced was subjected to real-time qPCR as above, using the primer/probe set designated ‘late’, and was normalised for input cDNA by primers and probe targeting GAPDH transcripts (300 nM forward primer GAPDH 5′- CCACATCGCTCAGACACCAT-3′,300 nM reverse primer GAPDH 5′-CCAGGCGCCCAATACG-3′, 150 nM probe GAPDH 5′-6-FAM-AGGTGAAGGTCGGAGTCAACGGATTTG-BHQ1-3′).
